# Spinal Subdural Hematoma in Ankylosing Spondylitis Patients Without Anticoagulation Therapy

**DOI:** 10.7759/cureus.48508

**Published:** 2023-11-08

**Authors:** Inka K Berglar, Gabriella P Williams, Joshua S Catapano, Laura A Snyder

**Affiliations:** 1 Department of Neurosurgery, Barrow Neurological Institute, St. Joseph's Hospital and Medical Center, Phoenix, USA

**Keywords:** spinal fracture, subdural hematoma, spine trauma, spinal subdural hematoma, ankylosing spondylitis

## Abstract

Patients with ankylosing spondylitis are at high risk of significant spinal trauma after relatively low-impact events, such as ground-level falls. Because of the osteopenic nature of the disease process, complex spinal fractures are common in these patients. Additionally, patients may sustain rare traumatic complications from these fractures, such as a spinal subdural hematoma (SSDH) or epidural hematoma. Traumatic SSDH is extremely rare, with few cases described in the literature, and cases are typically associated with antiplatelet or anticoagulant use. This study reviews the literature related to traumatic SSDH in patients with ankylosing spondylitis and describes two cases of traumatic SSDH in patients with ankylosing spondylitis without anticoagulation or antiplatelet therapy, which has not previously been reported in the literature.

## Introduction

Ankylosing spondylitis (AS) is a chronic inflammatory disease of the spine associated with ossification of the ligamentous apparatus, fusion of the vertebral joints, osteoporosis, and ultimately kyphosis [1]. The resulting remodulation of the spine increases its vulnerability to fractures and spinal cord injury.

Spinal subdural hematomas (SSDHs) are a rare cause of spinal cord injury. Although the pathophysiology of SSDH is debated, it has been associated with various causes that can be divided into three groups [2]: 1) posttraumatic [3]; 2) iatrogenic following spinal and cranial surgery [3-5], lumbar puncture [6], or spinal anesthesia [7,8]; and 3) spontaneous [9,10], including vascular malformations or tumors. Traumatic SSDH is extremely rare, with a few cases described in the literature, and it has typically been associated with anticoagulation use [7]. In this report, we describe two cases of traumatic SSDH in patients with AS without anticoagulation therapy, which to our knowledge has not previously been reported in the literature. We also review the literature related to traumatic SSDH in patients with AS.

## Case presentation

Case 1

**Figure 1:**
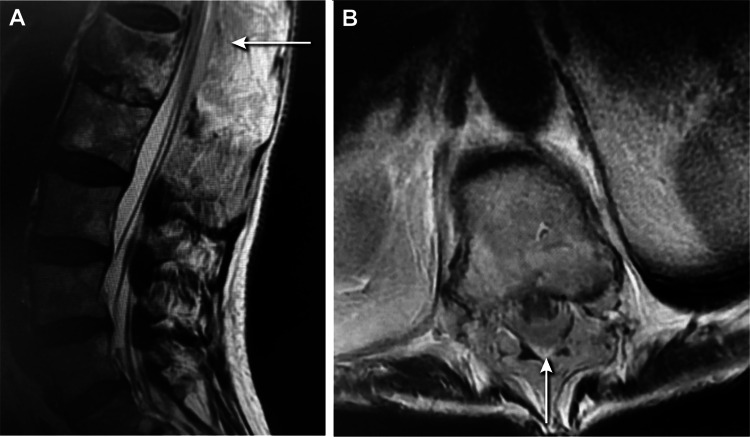
Preoperative MRI of the lumbar spine in case 1. Sagittal (A) and axial (B) preoperative imaging showing spinal epidural hematoma and spinal subdural hematoma (white arrows). Used with permission from Barrow Neurological Institute, Phoenix, Arizona.

A patient in their late 70s with no known medical history presented with severe low back pain and lower-extremity weakness five days after a mechanical ground-level fall onto the back. Motor examination findings included profound weakness in the bilateral lower extremities consistent with an American Spinal Injury Association impairment scale grade C spinal cord injury. Magnetic resonance imaging (MRI) findings included a three-column injury starting at L1 as well as epidural and subdural hematoma from T7 to L1, causing spinal cord compression (Figure 1).

The patient was not receiving any anticoagulation therapy. Imaging revealed typical signs of AS, including sclerosing ligaments, syndesmophyte formation, and a severe kyphotic deformity. The patient was taken to the operating room for T11-L3 fixation and fusion with T12-L2 laminectomies to evacuate the subdural hematoma. Postoperatively, the patient was transferred to the neurosurgery intensive care unit for monitoring. The patient had no complications associated with surgery. However, because of a poor baseline nutritional and cognitive status, after discussions with the family, the patient was eventually discharged to hospice.

Case 2

**Figure 2:**
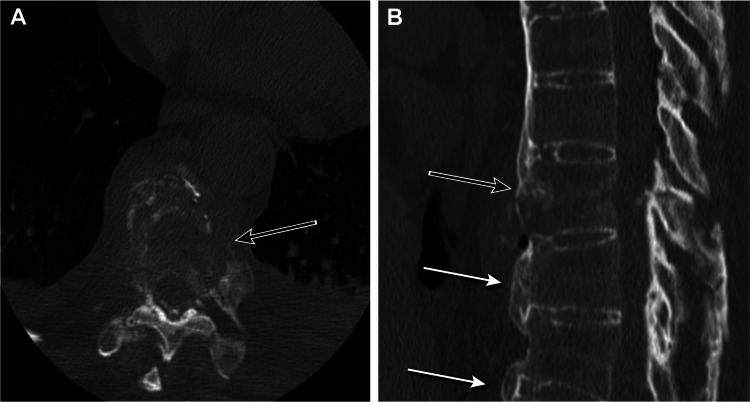
Preoperative computed tomography of the lumbar spine in case 2. Axial (A) and sagittal (B) preoperative imaging showing typical signs of ankylosing spondylitis (white arrows) as well as a T9 burst fracture (black arrows). Used with permission from Barrow Neurological Institute, Phoenix, Arizona.

A patient in their mid-70s with a history of type 2 diabetes presented to the hospital three days after sudden-onset back pain with profound bilateral lower-extremity weakness and bowel and bladder incontinence that began after lifting a heavy tire. Physical examination findings were consistent with the American Spinal Injury Association impairment scale grade C spinal cord injury, with 2/5 strength throughout the bilateral lower extremities and diminished rectal tone. Computed tomography revealed sclerosis of the ligamentous apparatus and syndesmophyte formation consistent with AS as well as a fracture at T9 concerning a three-column injury (Figure 2).

**Figure 3:**
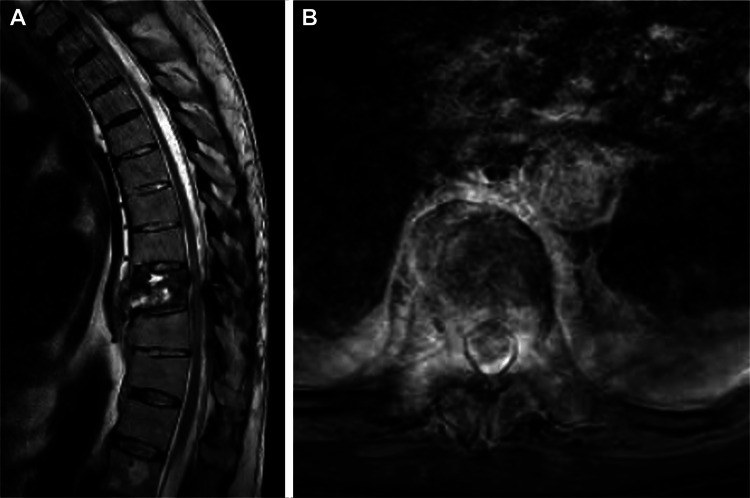
Preoperative MRI of the lumbar spine in case 2. Sagittal (A) and axial (B) imaging revealed spinal cord compression but no sign of spinal subdural hematoma. Used with permission from Barrow Neurological Institute, Phoenix, Arizona.

Subsequent MRI findings included severe canal stenosis secondary to retropulsion of the fractured T9 vertebral body as well as a compressive SSDH at that level (Figure 3).

The patient was taken to the operating room for T8-10 laminectomies and T7-11 posterior segmental fixation. During the decompression, a blue blush under the dura was noted, and the dura was subsequently opened for exploration. An SSDH was discovered on opening the dura, and it was subsequently evacuated. Postoperatively, the patient was transferred to the neurosurgical intensive care unit for close blood pressure monitoring, with a mean arterial pressure goal of >85 mm Hg for three days. Motor examination findings remained stable, with no changes from the finding of the immediate preoperative examination. The hospital stay was uneventful, and the patient was discharged to an acute rehabilitation facility on postoperative day 6.

## Discussion

AS is an important subtype of an interrelated group of rheumatic diseases, which are associated with various inflammatory processes causing back pain, oligoarthritis, and enthesitis. AS primarily affects the axial skeleton and sacroiliac joints and increases the rate of fractures up to fivefold compared to that in unaffected individuals [11]. Characteristic symptoms of AS include inflammatory back pain and spinal stiffness and immobility. Ultimately, osteoproliferation through syndesmophyte formation and ligamentous ossification lead to a rigid, brittle vertebral column that is prone to fractures [12]. For these reasons, AS patients are particularly vulnerable to severe spinal injuries following relatively minor traumas, such as a ground-level fall. Additionally, the risk for secondary neurological impairment due to traumatic spinal cord injury is increased in this patient population [1].

Most traumatic spinal hematomas are epidural [2] and originate from rupture of the vertebral venous plexus or are an extension of blood products from a vertebral fracture [1]. SSDHs account for only approximately 4% of cases of spinal hematomas. The mechanisms leading to traumatic SSDH are unclear. Some proposed mechanisms include trauma-induced rupture of subarachnoid veins that bleed into the cerebrospinal fluid space and expand into the subdural space by diffusion rupture of vessels located in the inner surface of the dura by an unneutralized force and subsequent pressure or transmission of subarachnoid blood to the subdural space through the thin arachnoid layer [13,14].

Treatment options vary between conservative and surgical management, and indications for surgical evacuation are dependent on the clinical context of the patient and the injury. In a meta-analysis of 122 patients with spontaneous SSDHs by de Beer et al. [2], 88 patients underwent surgery. That study reported that 50% of patients who underwent surgical decompression either fully recovered or were independent with respect to activities of daily living at the last follow-up [2]. Although an earlier report recommended decompression for SSDHs that are symptomatic or associated with neurological deficits [15], a recent literature review by Hsieh et al. [16] that reported 18 cases of traumatic SSDH suggests that conservative management may also be a reasonable treatment option for these patients. In that review, eight patients with isolated spinal trauma had associated SSDHs that were treated conservatively; of these patients, seven had improvement or complete resolution of their symptoms, including patients with profound motor and sensory deficits [16]. However, given the limited number of patients in that review, selection bias may have played an important role in the results. Ultimately, more data are needed to determine the optimal treatment for such patients.

Although SSDHs are generally extremely rare, they are thought to be more common in AS patients because of the ankylosing processes of the spine, which results in increased susceptibility to spinal cord injury and fractures even after minor trauma [17]. Previous studies have reported the incidence of spinal epidural hematomas (SEDHs) among posttraumatic AS patients to range from 20% to 50% [17], but a recent study showed that the incidences of SEDHs and SSDHs could be as high as 68% and 18%, respectively [18].

The rarity of these cases and the resulting small sample size contribute to selection bias, and the findings of these studies should be further evaluated. However, despite the rarity of SSDH, spine surgeons should be aware of the possibility of SSDH after minor trauma in AS patients and consider the need for possible surgical evacuation in addition to stabilization of any fractures.

## Conclusions

This is, to our knowledge, the first detailed report of two posttraumatic AS patients with SSDHs without prior anticoagulation therapy. Although the etiology of SSDH in patients with AS is not yet fully understood, these cases demonstrate that the appearance of SSDHs can be highly variable and can occur after only minor trauma in AS patients. Although SEDHs and SSDHs are extremely rare in general, AS patients are more likely than others to develop these hemorrhages. SEDH and SSDH should be considered as possible causes of spinal cord injury in this population and, if present, may require surgical evacuation.
